# Location method of airborne plant disease source based on a non-local-interpolation algorithm

**DOI:** 10.3389/fpls.2025.1553281

**Published:** 2025-05-23

**Authors:** Jing Zhang, Linglan Zhu, Yifang Wang, Si Chen, Yafei Wang, Shifa Li, Lu Xiao, Ning Yang

**Affiliations:** ^1^ School of Electrical and Information Engineering, Jiangsu University, Zhenjiang, China; ^2^ School of Innovative Design, City University of Macau, Macao, Macao SAR, China; ^3^ Fluid Machinery Center, Jiangsu University, Zhenjiang, China; ^4^ School of Agricultural Engineering, Jiangsu University, Zhenjiang, China

**Keywords:** Mie scattering theory, plant disease source localization, non-local diffusion simulation, power law equation, multi-sensor collaborative computing

## Abstract

The early stage pathogens of plant diseases have the characteristic of low concentration and difficult detection, which exacerbates the difficulty of tracing the disease, leading to rapid spread and difficulty in effective control. Currently, common plant disease detection techniques such as imaging and spectroscopy can only be applied after the occurrence and manifestation of diseases, and it is difficult to accurately locate the source of disease outbreaks during spore germination or propagation stages. Therefore, this paper proposes a method for locating the source of airborne plant diseases based on the non-local-interpolation algorithm. Firstly, a highly sensitive concentration sensor was designed based on Mie scattering theory to accurately count spores in plant diseases, and a multi-sensor collaborative computing network model was constructed. Secondly, by collecting spore quantity data at different locations, a particle diffusion model is established to summarize the propagation patterns of particles under specific regional conditions. Finally, a non-local-interpolation algorithm coupled with improved power-law equations was designed for precise localization of airborne plant disease sources under different wind and direction conditions. The experimental results in the greenhouse show that the maximum error of light scattering counting does not exceed 10%; Under windless and windy conditions, our method achieved localization accuracies of 94.7% and 92.9%, respectively, with approximately three nodes per square meter. This provides new ideas and insights for early diagnosis and precise localization of plant diseases.

## 1 Introduction

With the rapid development of computer technology, wireless sensor network technology has been widely used in the detection and identification of plant diseases, providing faster and more accurate diagnosis than traditional methods (Steeneken et al., 2023). However, the early detection of plant diseases is faced with severe tests. In the early stage of the disease, the concentration of pathogenic bacteria is low, which is difficult to detect, and the rapid spread of pathogenic bacteria through airflow further aggravates the complexity of disease tracing (Ali et al., 2022). Therefore, seeking new methods for early diagnosis and precise localization of plant diseases can help reduce agricultural economic losses, promote precise application of biologic agents, and reduce potential environmental impacts.

At present, biology-based methods for plant disease detection, such as polymerase chain reaction (PCR) method (Lal et al., 2023), quantitative real-time PCR method (Wang et al., 2023), and deoxyribonucleic acid (DNA) analysis (Chavhan et al., 2023), have become an efficient and accurate method of plant disease diagnosis. However, biology-based disease detection methods require a long time to complete sample collection, processing, and analysis (Yang et al., 2020), with weak timeliness, and cannot meet the needs of on-site real-time diagnosis. Image-based plant disease detection methods show significant timeliness (Li et al., 2024). They are often combined with machine learning algorithms to capture and preliminary analysis of the disease symptoms of plant leaves or tissues within a few seconds (Sajitha et al., 2024). (Liu et al., 2024) collected hyperspectral image data of virus-infected plant leaves and used machine learning algorithms for model training to establish an accurate model for identifying leaf disease areas, with a prediction accuracy of 97.5%. Although hyperspectral imaging technology can provide high-resolution spectral data and help in the detection of plant diseases, its high equipment cost and sensitivity to light intensity hinder its application in the field of rapid detection of plant diseases. (Panchal et al., 2023) collected image data of infected leaves and accurately labeled them according to disease characteristics. Then, key feature patterns were extracted from diseased leaf images through feature extraction and image segmentation, solving the problem of disease classification with an accuracy of 93.5%. However, in order to train highly accurate plant disease recognition models, it is necessary to rely on a large number of precisely labeled datasets (Yang et al., 2022). In addition, the image quality is easily affected by environmental lighting and background interference, and in the early stage of plant disease, because the symptoms are not obvious, and cannot be effectively recognized through images, resulting in lag.

In response to this issue, spore detection technology provides an effective solution. The pathogens of plant diseases will produce conidia under suitable temperature conditions, and spread to the surface of the plant through air flow (Yousef et al., 2024). Upon germination, these conidia form haustoria that penetrate plant cell surfaces, facilitating internal spread of the infection (Oro et al., 2018). Therefore, spore detection technology can achieve early warning by identifying the spores of pathogens before disease symptoms appear. In recent years, plant disease detection methods based on spore detection, such as spore capture apparatus (Kremneva et al., 2023) and microscopic image processing technology (Wei et al., 2024), have directly targeted the reproductive bodies of pathogens, providing direct evidence of disease occurrence, and can be detected in time even when the early symptoms of plant diseases are not obvious. (Mahaffee et al., 2023) collected spores in the air through a spore catcher, providing physical evidence for the early existence of pathogens. However, different species of spores may be so similar in appearance that spore capture apparatus have difficulty distinguishing between spores of similar morphology and size. (Zhang et al., 2024) through image processing technology, carried out detailed segmentation processing on the collect spore microscopic images to achieve the purpose of morphological recognition and counting of spores, and the evaluation indexes F1-score and mean intersection ratio (MioU) reached 0.943 and 0.925 respectively. The application of microscopic image processing technology frequently encompasses steps such as staining or surface modification, which may exert irreversible effects on the biomolecular structure or function of spores, and thus cause deviations in the diagnosis and classification of diseases. In contrast, light scattering technology provides a labeling-free spore detection method that can avoid these problems. By analyzing the scattered signals generated by the interaction between spores and light, (Liu et al., 2024) can conduct detection without disturbing the natural state of spores. (Son et al., 2023) developed a new method for monitoring plant diseases by utilizing the chemical specificity of scattered signals and combining them with sensor technology. Although light scattering technology can identify the presence of spores, it has not been able to achieve effective enrichment of spores and detection under real-time flow conditions, thus making it impossible to accurately locate plant diseases. (Lee et al., 2023) combined volatile organic compounds (VOCs) sensors and temperature and humidity sensors to achieve early detection of plant pathogens, and analyzed sensor data through a machine learning model for quantitative detection of viruses. It can be seen that single point detection cannot provide a detailed traceability analysis of the origin and transmission pathways of plant diseases.

The predominant modes of transmission for fungal diseases in greenhouse plants are air transmission, splash transmission, and seedling transmission (Spooren et al., 2024). When fungal diseases in greenhouse plants are transmitted through the air, the estimation of the location of the plant disease source region has a high degree of similarity with the location of air pollution sources. (Wang et al., 2021) proposed a reverse algorithm to estimate the quantity, location and intensity of unknown air pollution sources. (Kendler et al., 2021) deployed sparse sensor arrays to collect pollutant concentration data and then used an adaptive multi-objective evolution (MOEA) search algorithm to estimate source terms. However, although the transmission media of disease spores and air pollutants are the same, there are significant differences in the transmission mechanism between them. Because of their small size and mass, disease spores are relatively unaffected by Newtonian laws of mechanics. Their movement in the air is more likely to be affected by Brownian motion and airflow disturbance, which increases the uncertainty and complexity of the spore propagation path (Aliabadi et al., 2011). In contrast, although atmospheric pollutants may also be affected by airflow dynamics, their diffusion process is mainly regulated by physical processes and possibly accompanying chemical transformations (Pöschl, 2005). As a result, the atmospheric pollution source location model is not fully applicable to the spread analysis and location tracing of disease spores (Li et al., 2023).

Based on this, a method of locating airborne plant disease sources based on a non-local-interpolation algorithm was studied in this paper. Firstly, a highly sensitive concentration sensor was designed based on the Mie scattering theory to accurately count spores in plant diseases. Subsequently, by collecting spore quantity data at different locations, a particle diffusion model was established to summarize the diffusion patterns of particles under specific regional conditions. By analyzing the diffusion law, a non-local- interpolation algorithm coupled with improved power law equations was designed for precise localization of airborne plant disease sources under different wind and direction conditions. Our method provides a research solution for early diagnosis and traceability of plant diseases in the future.

## 2 Materials and methods

### 2.1 Experimental scenario setting

In this study, simulation experiments were carried out in a Venlo-type greenhouse with an area of 600 square meters. To mitigate environmental contamination, 5μm polystyrene nanoparticles were employed as surrogates for real botrytis spores, which typically range in size from a few microns to ten microns (Rhouma et al., 2022). The greenhouse was equipped with a wet curtain-fan forced cooling system. The average temperature inside the greenhouse was 21 ± 2 °C, and the relative humidity was 70 ± 10% RH. A microbial aerosol generator (TK-3) was employed to release 5μm nanospheres, with a concentration of 1mg/ml. The experimental nodes are arranged in a grid area of 0.8m*0.8m in the greenhouse to facilitate accurate control and monitoring. Since crops such as greenhouse strawberry cultivation are usually arranged in rows and planted in 20cm high ridges, their distribution can be approximated as a two-dimensional plane structure. Based on this geometric feature, the non-local- interpolation algorithm proposed in this paper is specially designed for two-dimensional space. In order to verify the accuracy of the simulation model, we set up 5 detection nodes in the experimental site to measure the nanosphere concentration and wind speed data to verify the consistency of the simulation results with the actual environment.

### 2.2 Method of disease spore counting

According to the size and arrangement of particles in the gas, the light scattering phenomena of the gas can be divided into different types and arrangement intervals, including Rayleigh scattering, Mie scattering, and Raman scattering (Kerker, 2013). The arrangement interval mainly depends on the density and spatial distribution of particles in the gas. For gases, Mie scattering typically occurs in small particles, such as water droplets, haze, and aerosols, with particle sizes ranging from a few nanometers to several hundred micrometers. These particles will produce obvious scattering effects on electromagnetic waves in visible and infrared bands. The formula is shown as Equation 1 (Jones, 1999):


(1) $$\sigma_{SC}=\frac{2\pi }{k^{2}}\sum_{n=1}^{\infty }\left(2n+1\right)|\frac{a_{n}}{b_{n}}{|}^{2}$$

where *σ_SC_* is the scattering cross-sectional area; *k* is the wave number of the incident wave, *k*=2*π/λ*, and *λ* is the wavelength of the incident wave; *ɑ_n_* and *b_n_* are the Mie coefficients, which represent the amplitudes of the electric and magnetic fields of the electromagnetic wave on the surface of a particle, and are at the heart of the Mie scattering theory.

**Figure 1:**
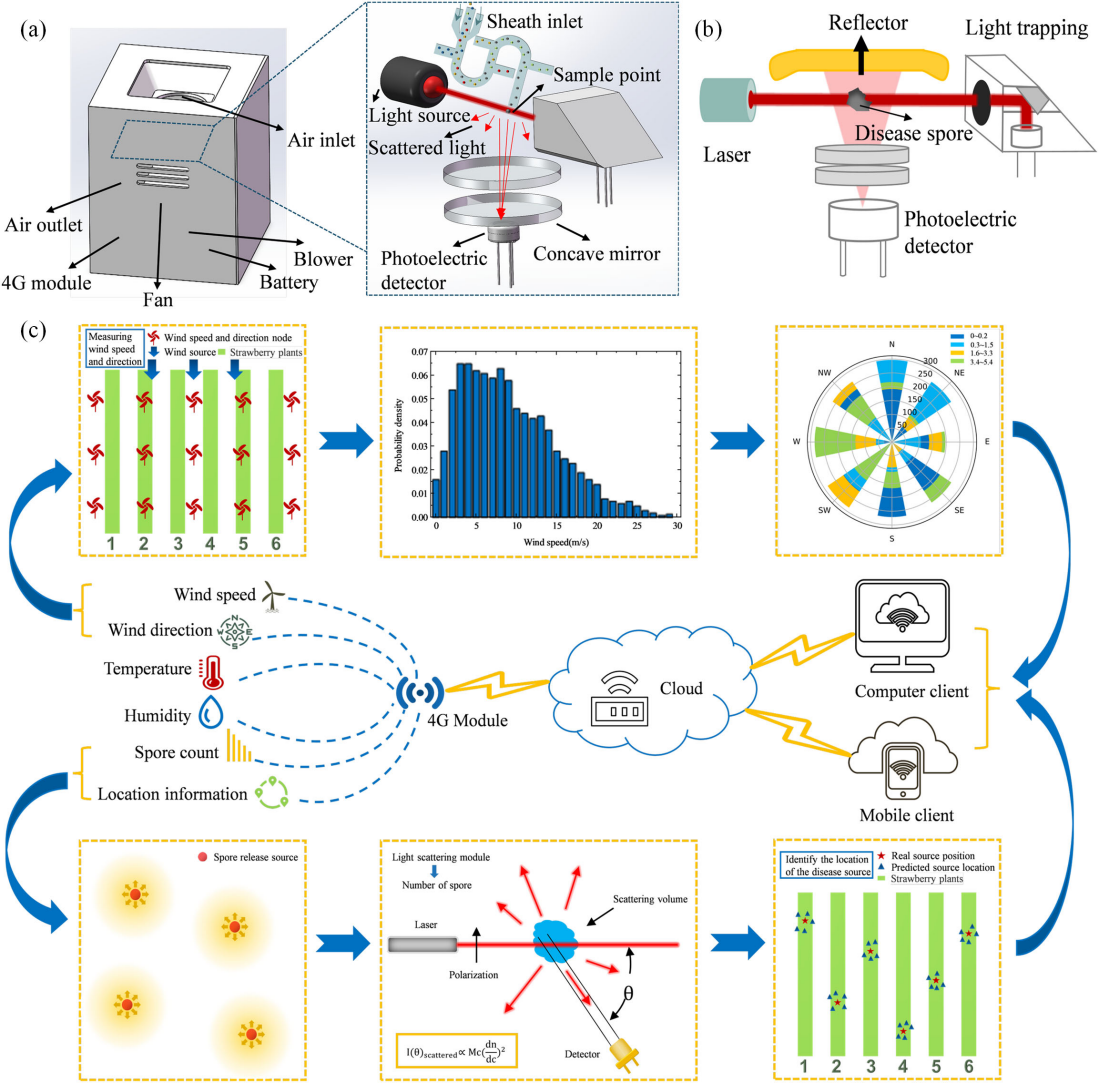
Schematic diagram of airborne plant disease location based on non-local-interpolation algorithm. **(a)** Node shell and Light scattering structure. **(b)** Light scattering schematic diagram. **(c)** System frame diagram.

The particle size range of plant disease spores is usually between a few microns and tens of microns, as shown in **Table 1** (Wang et al., 2022), which is equivalent to the wavelength of visible light, so the Mie scattering theory can be considered to count disease spores. We use a laser with the same wavelength as the characteristic of plant disease spores to illuminate the collected disease spores to produce scattering phenomena. By collecting scattered light signals at a specific angle, the curve of scattering light intensity with time can be obtained. Subsequently, the microprocessor employs an algorithm based on Mie scattering theory to analyze the intensity and angle of scattered light, thereby calculating the refractive index and extinction coefficient of particles (Shao et al., 2017). This analysis enables a certain degree of differentiation between spores and inorganic particles to infer the number of different disease spores per unit volume. In order to improve the accuracy of distinguishing between spores and inorganic particles, we introduce cardinal subtraction. The background particle concentration was determined using a light scattering sensor in an environment devoid of plant disease spores and was recorded as a baseline. Whereafter, this baseline is then subtracted from the particle concentration measured in the actual environment. This subtraction effectively mitigates the interference from environmental background noise, thereby yielding a more accurate determination of the actual concentration of plant disease spores. The sensor structure is shown in **Figures 1a, b**. The scattering module consists of a 635nm wavelength laser, focusing lens, reflector, photomultiplier tube, light trap, and gas channel.

**Table 1:** Average spore size of fungal and bacterial diseases of major greenhouse crops.

Species	Spore size (μm)
B. cinerea spores	19.3 (11.4–26.7) × 11.7 (8.3–14.5)
P. cubensis spores	30.6 (21.1–39.8) × 20.5 (13.8–23.6)
P. xanthii spores	35.4 (30.2–39.5) × 14.2 (7.3–22.2)

### 2.3 Location network construction and framework of IoT node

The framework of airborne plant disease source location method based on non-local-interpolation algorithm proposed in this paper is shown in **Figure 1c**. Firstly, the cloud server receives the spore count information sent by the node. Secondly, the cloud server receives the temperature and humidity information and wind speed and direction information sent by the node. Then, according to the analysis of spore number data and environmental information, the location information of plant disease source was obtained by using disease source location algorithm. Finally, the cloud platform synchronizes the number of spores and the location of plant disease sources to the client.

The node takes MCU module (STM32F407VGT6) as the core to realize data collection and transmission. The concentration sensor based on laser scattering has a detection accuracy of 90%. The detection range and accuracy of the temperature and humidity sensor are -40 to 100°C (± 1°C) and 0 to 100% RH (± 3% RH), respectively. The wind speed and direction sensor (SM5388V) has a wind speed detection range and accuracy of 0~30 m/s (± 3%), and a wind direction detection range and accuracy of 0~360° (± 22.5°). Each detection node is equipped with a 4G module (EC20), which regularly transmits environmental information data to the cloud server, and the cloud server can also send commands to the node for data collection. We use Alibaba Cloud Internet of Things as a cloud server, these nodes through the 4G network to achieve data storage and circulation. In addition, each detection node is equipped with a battery (12 V) to supply power.

### 2.4 Construction of diffusion model

In order to accurately analyze the flow field in the greenhouse, we used SolidWorks to accurately model the experimental area and SolidWorks Flow Simulation to conduct gas diffusion analysis (Fedorenko et al., 2023). Taking into account the requirements of changing flow field gradients, we used an unstructured meshing approach and refined the area near the wet curtain and the fan perimeter to ensure better flow detail capture.

In the experimental environment, gas flow and heat exchange follow the basic principles of conservation of mass, momentum, and energy (Bejan, 1987). Considering the low airflow speed in the greenhouse, this case can be described as a low Reynolds number flow, and the compressibility of the fluid is usually negligible (Bartzanas et al., 2013), thus simplifying the analysis process. Nevertheless, the physical properties of the fluid, such as density and viscosity, exhibit variations over time and space. Our simulation comprehensively accounts for and reflects these changes. When simulating the greenhouse flow field, the Euler method was used to numerically describe the motion characteristics of the flow field, so as to obtain the distribution of state parameters such as fluid velocity, pressure, and temperature (Chen et al., 2006). Through detailed modeling of gas flow and heat exchange processes in the greenhouse, we can provide a reliable basis for experimental design and equipment optimization (Zhang et al., 2021).

In addition, in order to better describe the turbulence characteristics of greenhouse air and optimize the experimental environment. We choose to adopt the standard *k*-*ϵ* model, which is one of the most widely used turbulence models in computational fluid mechanics and can effectively predict the development and transfer process of turbulence (Yu and Thé, 2016). By combining the actual situation of the greenhouse with the standard *k*-*ϵ* model, we can better understand the characteristics of the air flow in the greenhouse, including the formation, transmission and attenuation of turbulence, which helps to improve the efficiency and reliability of the experiment. In the standard *k*-*ϵ* model, *k* and *ϵ* are unknown quantities. The *k* equation and the *ϵ* equation are expressed as Equation 2 (Shaheed et al., 2019):


(2) $$\begin{array}{l} \frac{\partial k}{\partial t}+\frac{\partial (u_{i}k)}{\partial x_{i}}=\frac{\partial }{\partial x_{i}}(Dk_{eff}\frac{\partial k}{\partial x_{i}})+G_{k}-\epsilon \\ \frac{\partial \epsilon }{\partial t}+\frac{\partial (u_{i}\epsilon )}{\partial x_{i}}=\frac{\partial }{\partial x_{i}}(D\epsilon_{eff}\frac{\partial \epsilon }{\partial x_{i}})+C_{1\epsilon }\frac{\epsilon }{k}G_{k}-C_{2\epsilon }\frac{\epsilon^{2}}{k} \end{array}$$

Together, these equations describe the transport and evolution of turbulent kinetic energy *k* and turbulent dissipation rate *ϵ* in a fluid.

To determine the fluid temperature distribution influenced by diverse thermal sources, which subsequently modulates the fluid’s viscosity and density, and thereby governs its flow and diffusion dynamics, the energy equation is utilized to characterize the conservation of thermal energy within the fluid. The equation is presented as Equation 3 (Antontsev et al., 2002):


(3) $$\rho c_{p}(\frac{\partial T}{\partial t}+u\cdot \nabla T)=\nabla \cdot (k\nabla T)+Q$$

where *c_p_* is the specific heat capacity; *T* is the temperature; *k* is the thermal conductivity and *Q* is the heat source term.

### 2.5 Disease location algorithm

**Figure 2:**
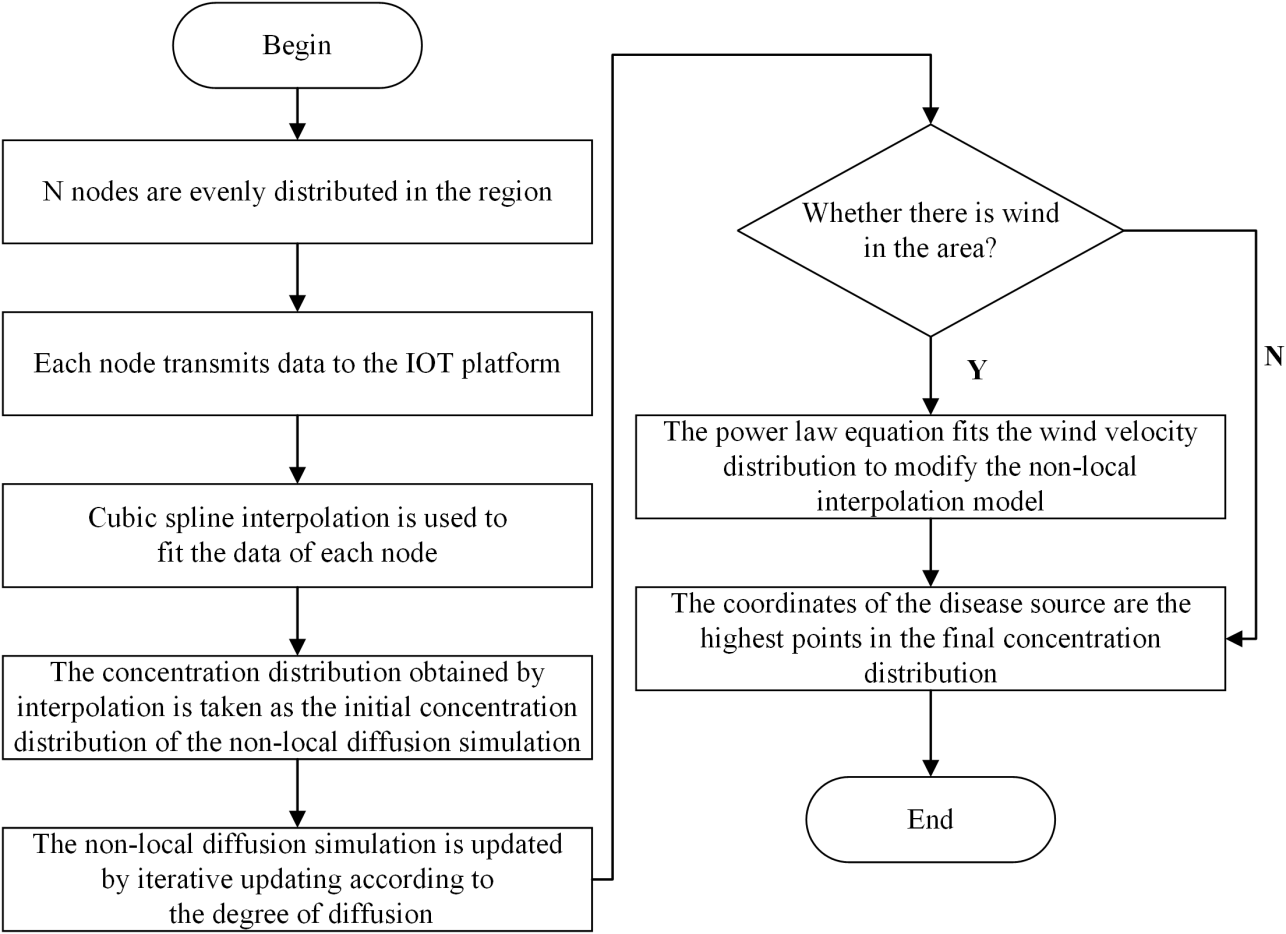
Flow chart of non-local interpolation localization algorithm.

Considering the impact of disease spores on the environment, we chose nano-microspheres instead of real spores for the experiment. Each node in the wireless sensor network monitors the concentration of nanospheres in the current environment. According to the coordinates of the nodes and the observed concentration data of the nanospheres, the concentration distribution is obtained by cubic spline interpolation. This concentration distribution was used as the initial concentration distribution of the non-local diffusion simulation, and then the non-local diffusion simulation was carried out by iterative updating to obtain the final concentration distribution, so as to estimate the disease source. We considered the localization algorithm of nanospheres in both windless and windy conditions. The windless state refers to the diffusion of particles not being affected by the airflow field. The flowchart of the non-local-interpolation localization algorithm is shown in **Figure 2**.

The formula of the non-local-interpolation algorithm is as Equation 4:


(4) $$\left\{ \begin{array}{l} C_{0}(x,y)\\ \frac{\partial C}{\partial t}=D\nabla^{2}C\\ B=f(v) \end{array}\right.$$

where *B* is the boundary condition, *C* is the concentration of the substance, *t* is the time, *D* is the diffusion coefficient, and 𝛁^2^ is the Laplace operator, which represents the spatial variation of concentration. In this case, assume that there is a set of known data points (*x_i_,y_i_*), where *i*=0,1,2,……,*n*, and satisfy *x*_0_<*x*_1_<*x*_2_<…<*x_n_*. *A* smooth curve *C*_0_(*x*,*y*) is generated through these points.

Fit a cubic polynomial between each neighboring data point to obtain a series of cubic polynomial segments. For the *ith* data point, assume that the cubic polynomial is *S_i_* (*x*), where *x* falls in the interval [*x_i_,x_i_*_+1_]. This cubic polynomial can be expressed as Equation 5:


(5) $$S_{i}(x)=a_{i}+b_{i}(x-x_{i})+c_{i}{\left(x-x_{i}\right)}^{2}+d_{i}{\left(x-x_{i}\right)}^{3}$$

The coefficients of the cubic polynomial are determined by the Equation 6 steps:


(6) $$\left\{ \begin{array}{l} S_{i}(x_{i})=y_{i}\\ S_{i}^{'}(x_{i+1})=S_{i+1}^{'}(x_{i+1})\\ S_{i}^{''}(x_{i+1})=S_{i+1}^{''}(x_{i+1}) \end{array}\right.$$

The non-local diffusion term encompasses both horizontal and vertical diffusion functions. Given that the nodes data were collected along the same horizontal plane as the plant, this study focuses solely on the horizontal diffusion function. The distinction among the three prevalent single-parameter models lies in the characteristics of their tails. The tail quality of the horizontal propagation function is a critical aspect in plant epidemiology, as it influences the pattern of epidemic spread. The distributions are ranked from the lightest to the heaviest tail: Gaussian distribution, Exponential distribution, and Cauchy distribution function, respectively (Soubeyrand et al., 2008). Given the enclosed nature of a greenhouse, which tends to restrict long-range propagation, Gaussian or Exponential distributions are deemed more suitable for modeling. Furthermore, the high humidity and optimal temperatures prevalent in greenhouses can enhance the transmission of diseases. Consequently, distributions with heavier tails, such as the Exponential or Cauchy distributions, are more frequently utilized. After careful consideration, we have selected the product of the Exponential distribution and the initial concentration distribution to represent the non-local diffusion term.

In greenhouse simulations, it is often assumed that the walls and tops of the greenhouse are impermeable and that the formula for the concentration of diffusive substances at the boundaries is as Equation 7:


(7) $$\left\{ \begin{array}{l} f(v)=0,v=0m/s\\ f(v)=v(x,y),v\neq 0m/s \end{array}\right.$$

The improved power-law equation is as Equation 8:


(8) $$v(x,y)=k\cdot (x_{\;}^{a}\cdot y_{\;}^{b})$$

where *v*(*x*,*y*) is the wind speed at coordinates (*x*,*y*), *k* is constant, *ɑ* and *b* are exponents. In the experimental environment of this study, when the wind speed is 1m/s, *k*=0.29591, *ɑ*=0.11069, *b*=0.14347.

Finally, a final concentration distribution is obtained based on the results of the non-local diffusion simulation. This distribution will take into account the initial predicted distribution and the effects of the diffusion process.

## 3 Results and discussion

### 3.1 Verification of the light-scattering nodes

**Figure 3:**
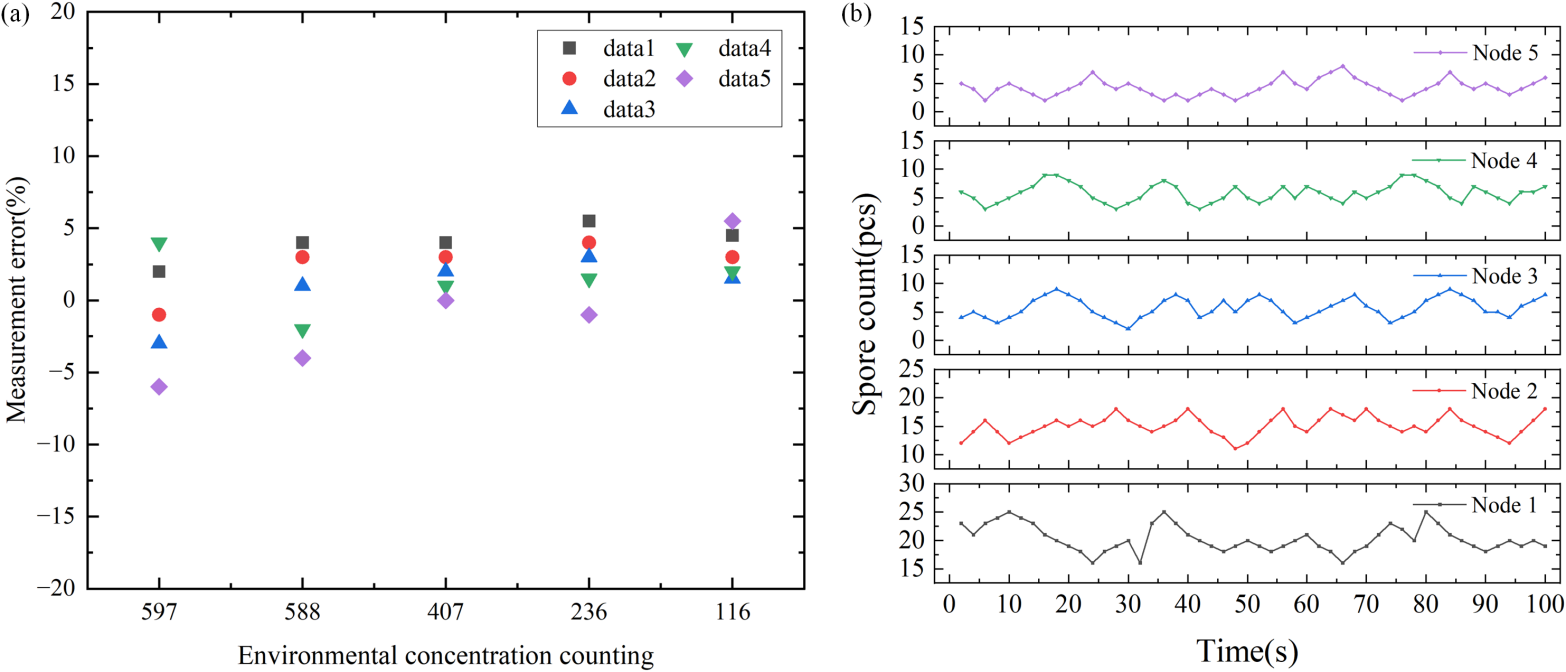
Laser scattering node test results. **(a)** Normal temperature consistency error distribution of light scattering nodes. **(b)** Spore count at five different locations at the same time.

To verify the detection performance of the laser scattering node for particulate matter, the nodes was tested at the ambient temperature of 21±2 °C and the ambient humidity of 70±10% RH, using 5μm nanospheres as the release source. The test results are shown in **Figure 3a**, indicating that the actual consistency error at room temperature is ±10%. Compared with ordinary laser scattering sensors, it has higher consistency and accuracy. In addition, we positioned node 1 near the visually identified gray mold area in the strawberry greenhouse, while nodes 2, 3, 4, and 5 were distributed around it. In the case of subtracting the environmental base, it is obviously found that the value of node 1 is higher than that of other nodes, thus confirming that this node can effectively detect the presence of disease spores, as shown in **Figure 3b**.

### 3.2 Diffusion model verification and wind field distribution verification

Since there is always airflow in the greenhouse, the particles always follow a non-Gaussian distribution. In windy conditions, particles travel through the environment with airflow, and their diffusion path and range are significantly affected by wind speed and direction. Our research not only covers the diffusion behavior of particles under constant wind speed conditions, but also extends to the effects of temperature and humidity on the diffusion of nanospheres.

**Figure 4:**
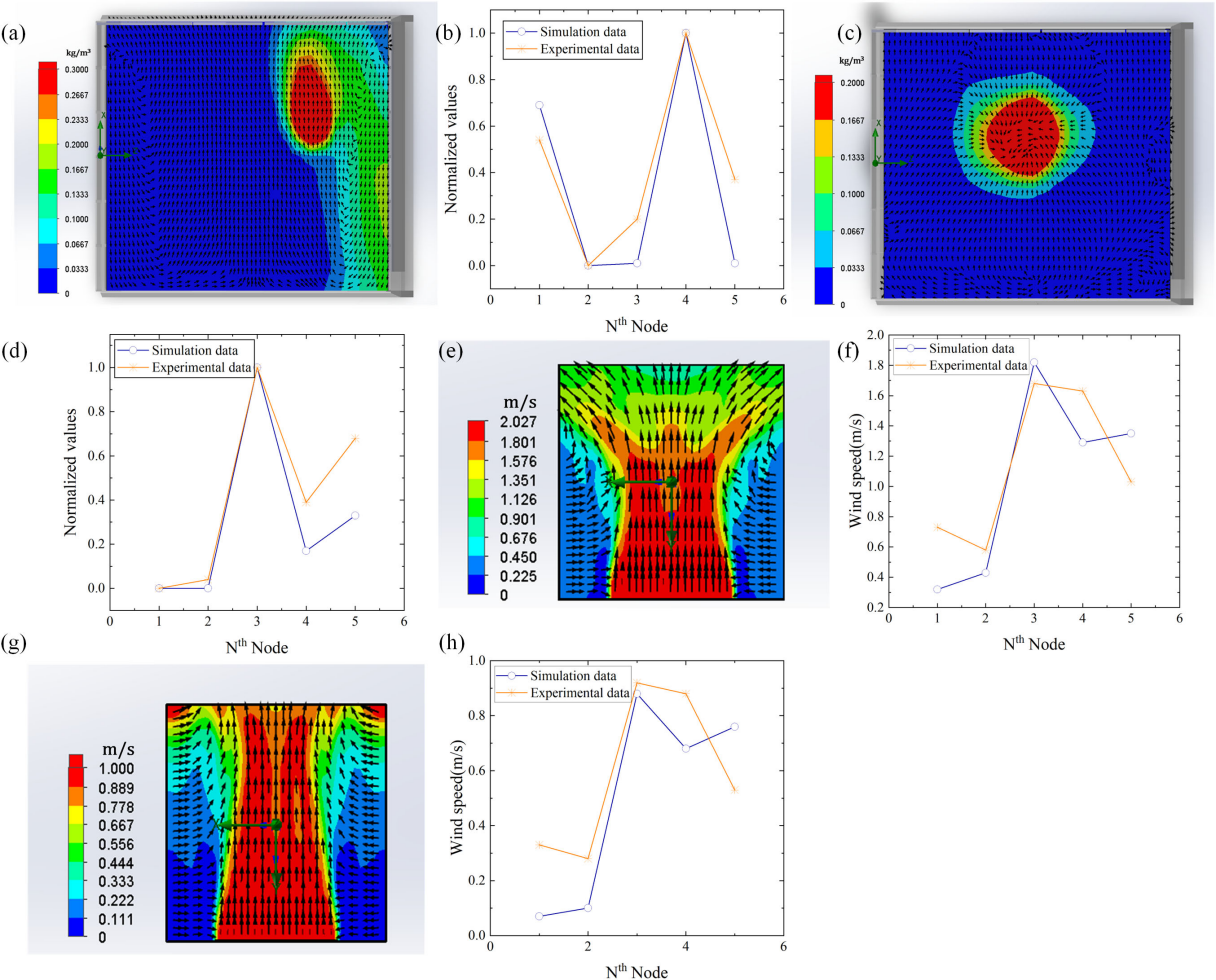
Comparison diagram between simulation and experiment. **(a)** Diffusion simulation under windy conditions. **(b)** Normalization comparison between simulated data and experimental data under windy conditions. **(c)** Diffusion simulation under windless conditions. **(d)** Normalization comparison between simulated data and experimental data under windless conditions. **(e)** Wind speed simulation at 2 m/s. **(f)** Comparison of simulation and experimental data at 2 m/s wind speed. **(g)** Wind speed simulation at 1 m/s. **(h)** Comparison of simulation and experimental data at 1 m/s wind speed.

To verify the accuracy of the simulation model, a microbial aerosol generator (TK-3) and nanospheres (5μm) were used as particle release sources. Five detection nodes were arranged at the sites to measure the concentration of the nanospheres, and the diffusion of the nanospheres was verified by using a fan to simulate natural wind. SolidWorks Flow Simulation software was used to draw the diffusion diagram of nanospheres and analyze the diffusion trend of nanospheres. As shown in **Figure 4a**, when there is wind, the distribution of nanospheres will have a greater diffusion in the downwind direction. Due to the environmental pressure where the wet curtain is set, the nanospheres may spread to the area with lower pressure based on the wind direction, resulting in a certain diffusion of the nanospheres on the side. As shown in **Figure 4c**, the distribution of the nanospheres showed a tendency to spread from the center to the periphery under the condition of windless. To further evaluate the accuracy of the simulation model, we normalized the nanosphere concentration data at 5 different locations measured by the experiment and compared them with the normalized simulation data. The results show that there is a high correlation between the experimental data and the simulation data, with correlation coefficients of 0.9144 and 0.9280 in windy and windless conditions, respectively shown in **Figures 4b, d**.

In the environment under the rotation of the greenhouse fan, most plants require the air passing speed between 1m/s and 2.78m/s to ensure that the growth and ventilation needs of the plant are met. Therefore, under normal circumstances, when the fan in the greenhouse is turned on, the airflow speed should be maintained within this range to ensure that it will not adversely affect the plants, but also effectively carry out ventilation. By using SolidWorks modeling and Flow Simulation analysis, we simulated the wind field distribution of 2 m/s in a specific area and compared the simulation data with the experimental data after normalization, as shown in **Figure 4e**. **Figure 4f** shows that the correlation coefficient results in 0.8796. Similarly, we simulated the wind field distribution in a specific region at 1m/s wind speed and compared it with the actual measured data after normalization, and the correlation coefficient was 0.8633, as shown in **Figures 4g, h**.

### 3.3 Plant influences in experimental settings

**Figure 5:**
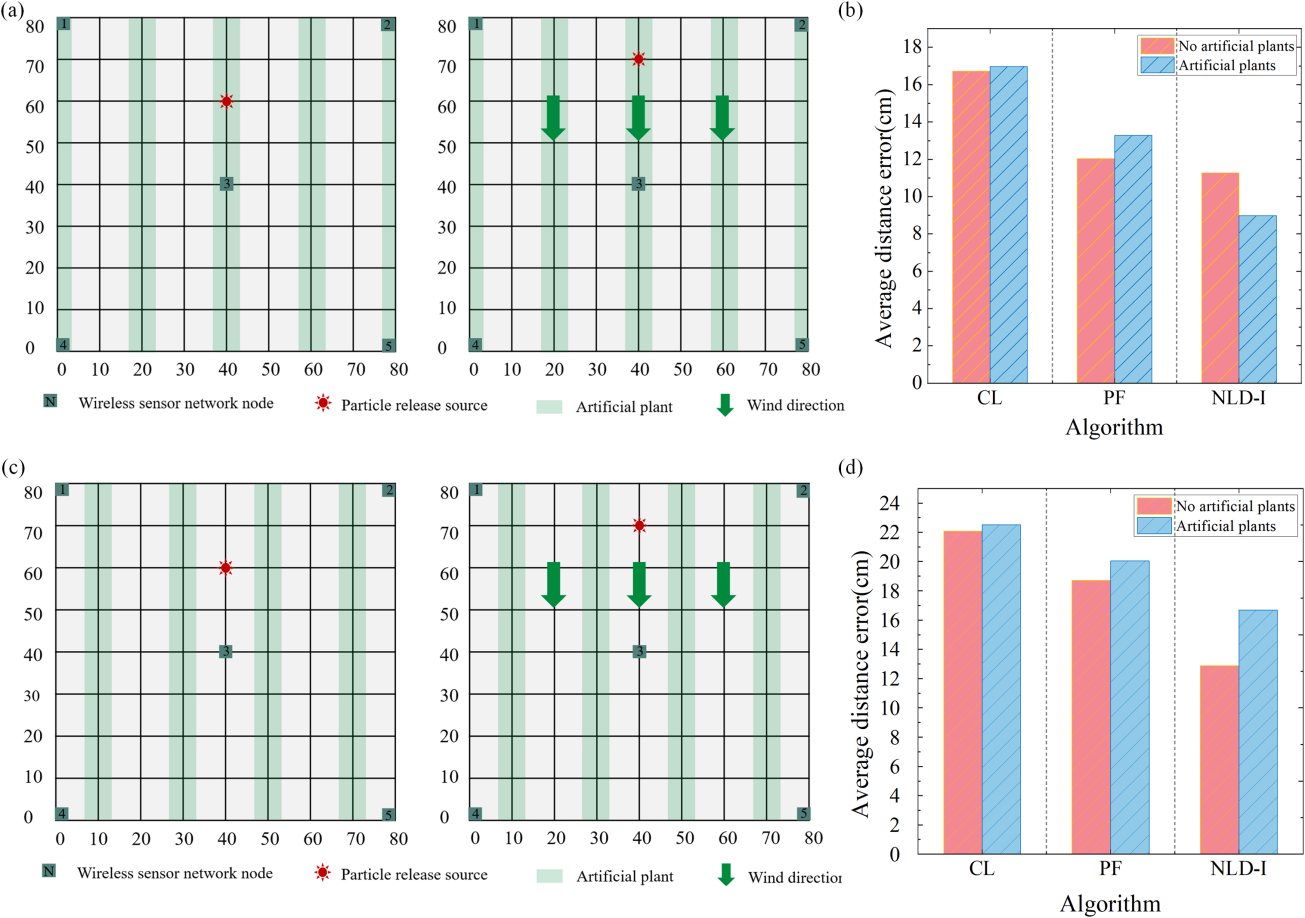
Effect of plant disturbance on sensor deployment. **(a)** Nodes are deployed between plants. **(b)** Comparison of positioning errors of the three algorithms with or without plant interference when the wind speed is 0 m/s. **(c)** Nodes are deployed next to the plants. **(d)** Comparison of positioning errors of the three algorithms with or without plant interference when the wind speed is 1 m/s.

In the process of node deployment of wireless sensor networks, many factors need to be considered, including signal transmission range, signal interference, energy consumption and data acquisition efficiency. When the sensor node is deployed in the plant, it may encounter problems of occlusion and interference caused by the plant itself. This physical barrier can lead to instability in signal transmission, which in turn affects the accuracy and reliability of the data. The sensor nodes deployed near the plant can effectively reduce the signal transmission interference caused by the plant. This layout helps to improve the signal stability and the overall performance of the network. Artificial plants are used in this experiment. **Figure 5a** shows the layout of sensor nodes deployed inside the plant; **Figure 5c** shows a layout scheme where sensor nodes are deployed next to the plant.

To systematically evaluate the performance differences of two different node deployment schemes in particle release source localization experiments. In this study, we designed the following experiment, in which the particle release source was randomly placed in five different locations, and the test was repeated three times at each location. Under the conditions of windless and windy, the influence of plant interference on sensor nodes on positioning accuracy is shown in **Figures 5b, d**, respectively. The results show that, overall, the plants have a significant interference effect on the data obtained by the sensor, and in the evaluation of the localization algorithm, the non-local-interpolation (NLD-I) algorithm shows higher adaptability than the particle filter (PF) algorithm and the centroid localization (CL) algorithm. We deployed sensors in a 600 square meter greenhouse using the strategy depicted in **Figure 5c**. The distribution density was calculated by dividing the area of a circle defined by the positioning error radius by the total experimental area, yielding an average density of approximately 3 sensors per square meter.

### 3.4 Positioning algorithm verification

**Figure 6:**
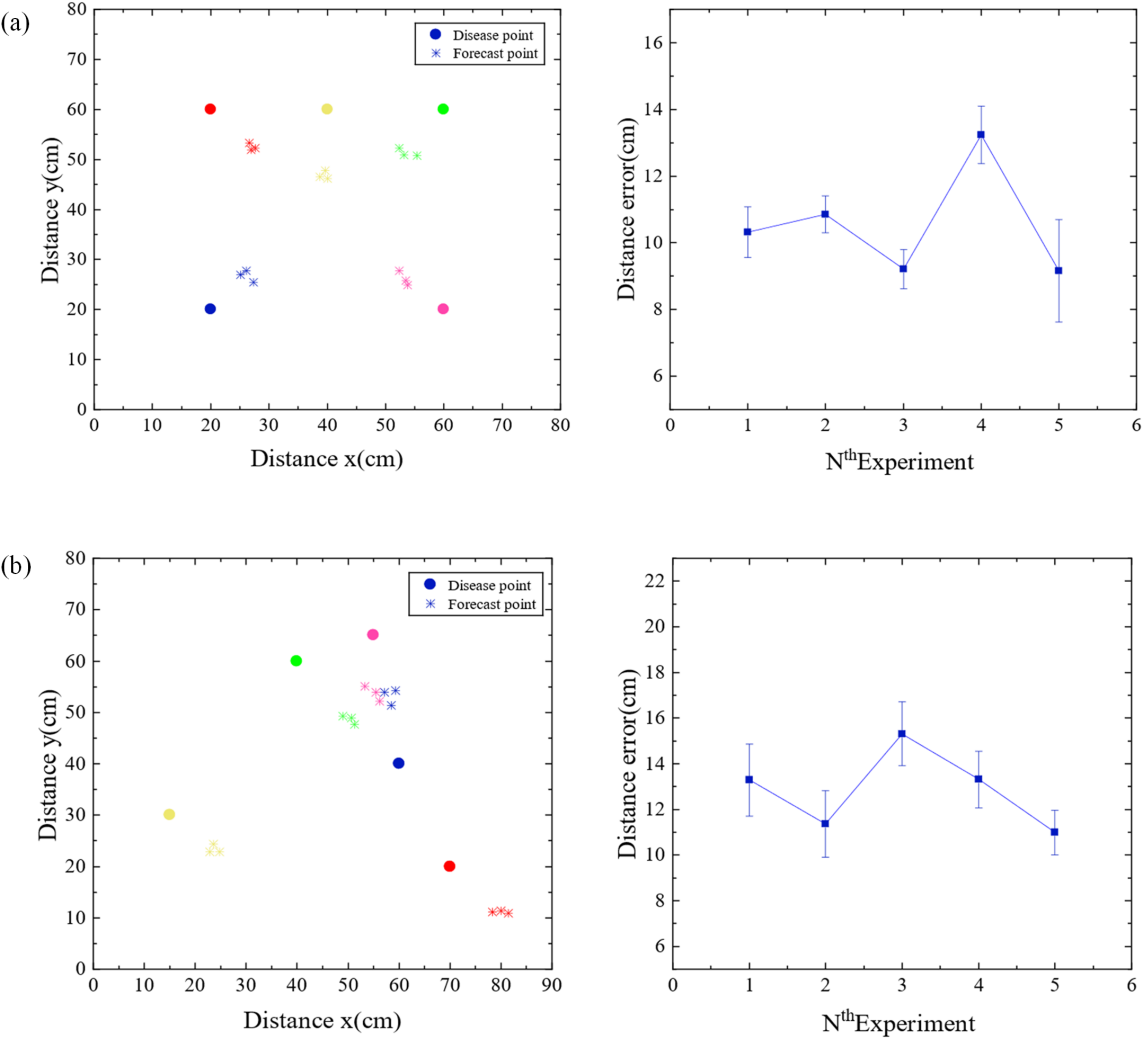
Positioning error diagram. **(a)** The location distribution map and location error diagram of non-local interpolation algorithm when there is no wind. **(b)** The location distribution map and location error diagram of non-local interpolation algorithm when the wind is 1 m/s.

**Figure 7:**
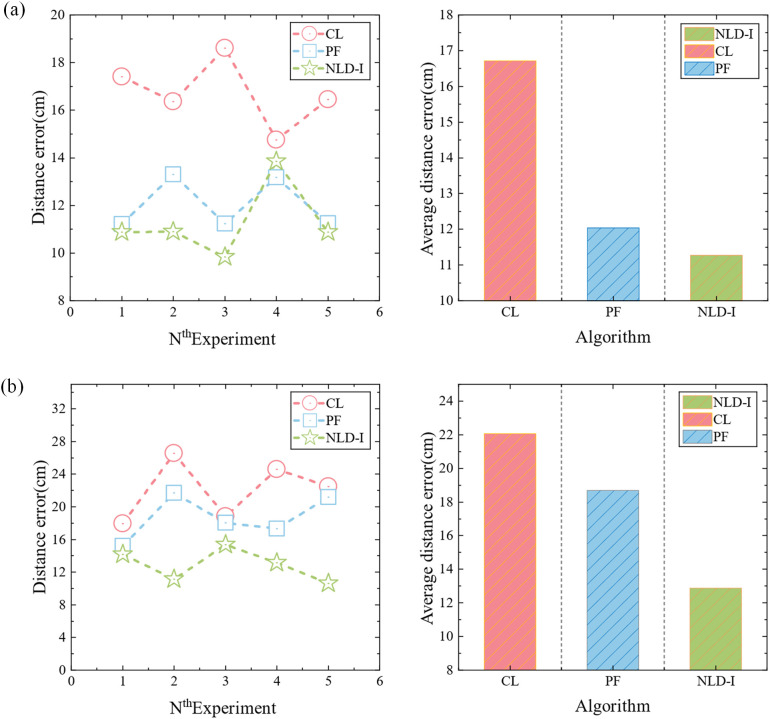
Positioning error comparison diagram. **(a)** Comparison of positioning errors and comparison of the average positioning errors of the three algorithms when there is no wind. **(b)** Comparison of positioning errors and comparison of the average positioning errors of three algorithms when the wind speed is 1 m/s.

In order to accurately predict the location of pathogen of strawberry gray mold, a disease spore transmission model was established according to the transmission characteristics of disease spores in windy and windless conditions. Cubic spline interpolation algorithm, non-local diffusion simulation, and improved power-law model were used to predict the location of the disease source. To verify the accuracy of the positioning algorithm, the microbial aerosol generator (TK-3) was first used as the particle release source, and the pre-equipped nano microspheres solution was continuously sprayed, and five nodes were arranged according to **Figure 5c**. The nodes were composed of light scattering module, Internet of Things module, battery, and MCU module. Then five different positions were randomly selected for the experiment, and each position was tested three times. The diffusion of the nanospheres was verified by using a fan to simulate natural wind. When there is no wind, the non-local-interpolation algorithm is used to locate the release source, and the positioning error is shown in **Figure 6a**. Under windy conditions, improved power-law equation model was introduced into the non-local-interpolation algorithm to locate the release source. The positioning error when the wind speed was 1m/s is shown in **Figure 6b**. In addition, under the same environmental interference condition, the comparison of positioning errors of the non-local-interpolation algorithm (NLD-I), the centroid positioning algorithm (CL) and the particle filter algorithm (PF) under the conditions of windless and windy is shown in **Figures 7a, b** respectively. Therefore, compared with the non-local-interpolation algorithm, the particle filter algorithm has the disadvantages of randomness, high computational complexity, and difficult particle number selection, while the centroid location algorithm is not sensitive to small data concentration changes. By taking the positioning error distance as the radius, the positioning accuracy of the non-local-interpolation algorithm is 94.7% and 92.9%, respectively, under the conditions of windless and windy.

### 3.5 Comparative analysis

In order to verify the superiority of the airborne plant disease source localization method designed in this study, we comprehensively compared its concealment, wind speed interference resistance, real-time on-site detection ability, cost-effectiveness, and non-destructive testing characteristics with existing plant disease detection methods in the early stages of the disease. The results are listed in **Table 2**. (Chavhan et al., 2023) proposed a novel approach for the early detection of plant diseases based on multiple molecular marker-assisted analysis. This method encompasses both multiplex PCR detection and CAPS labeling, characterized by its high specificity and sensitivity. Nevertheless, this method is lossy detection and difficult to detect in real time in the field. (Panchal et al., 2023) employ an image-based deep learning approach for plant disease detection. This method requires labeling of leaf images of infected crops according to disease patterns and utilizing pixel-based operations to enhance the information in the images. Then feature extraction and image segmentation were carried out, and crop diseases were classified according to the extracted diseased leaf patterns. But in the absence of obvious symptoms, this method cannot detect early plant diseases. (Xie et al., 2024) proposed a plant disease detection method based on hyperspectral imaging technology. This method used hyperspectral imaging technology to detect plant biological stress, thus achieving early diagnosis. However, hyperspectral cameras and related equipment are relatively expensive. Above analysis shows that the plant disease detection method proposed in this paper can timely diagnose the disease and locate the source of the outbreak in the early stage of plant disease, with strong anti-interference ability and cost-effectiveness. Therefore, this method has application potential and practical value in the field of plant disease detection.

**Table 2:** Comparison between the proposed method and the current methods of plant disease detection.

Reference	No obvious symptoms in the early stage of plant disease	Resistance to wind interference	On-site real-time detection capability	Cost-effectiveness	Nondestructive testing characteristics
(Chavhan et al., 2023)	✓	✓	×	✓	×
(Panchal et al., 2023)	×	✓	✓	✓	✓
(Xie et al., 2024)	✓	×	✓	×	✓
proposed	✓	✓	✓	✓	✓

## 4 Conclusion

In this paper, a method for locating airborne plant disease sources based on non-local-interpolation algorithm is proposed. Firstly, the scattering characteristics of spores are studied, and a high-sensitivity concentration sensor is designed on the basis of Mie scattering theory. The concept of radix subtraction is innovatively introduced to accurately count spores in plant diseases. Secondly, two sensor deployment schemes are designed, the optimal sensor deployment strategy is determined by applying the accurate positioning algorithm, and the wireless sensor network model is constructed. Then, by collecting data on the number of spores at different locations, a particle diffusion model was established to deeply study the propagation law of particles under specific regional conditions, and simulation experiments were carried out through SolidWorks flow simulation software. The correlation coefficients between the particle diffusion predicted by the simulation model and the experimental data reached 0.9144 and 0.9280 respectively under windy and windless conditions. Finally, according to the law of particle propagation, a disease source location algorithm coupled with the non-local-interpolation algorithm and improved power-law equation was designed to accurately locate the plant disease source under different wind direction conditions. The results of greenhouse experiments show that our method achieves localization accuracy of 94.7% and 92.9% respectively under windless and windy conditions with approximately three nodes per square meter. In summary, the source location method of airborne plant diseases based on the non-local-interpolation algorithm can realize the early diagnosis and location tracing of plant diseases.

## Data Availability

The datasets presented in this article are not readily available because Data were measured on the spot in the experimental setting of this study and no public data set was generated. Requests to access the datasets should be directed to Linglan Zhu, zll1069137969@163.com. The proposed method achieved the well performances in early diagnosis and source tracing of plant diseases, and all its source codes are publicly available at https://anonymous.4open.science/r/test-7023.
